# Childhood‐onset cerebellar ataxia in Japan: A questionnaire‐based survey

**DOI:** 10.1002/brb3.1392

**Published:** 2019-08-30

**Authors:** Hiroya Ono, Yuko Shimizu‐Motohashi, Kazushi Maruo, Eri Takeshita, Akihiko Ishiyama, Takashi Saito, Hirofumi Komaki, Eiji Nakagawa, Masayuki Sasaki

**Affiliations:** ^1^ Department of Child Neurology National Center Hospital for Neurology and Psychiatry, NCNP Tokyo Japan; ^2^ Department of Biostatistics Faculty of Medicine University of Tsukuba Ibaraki Japan

**Keywords:** autosomal dominant ataxia, childhood‐onset, dentatorubropallidoluysian atrophy, questionnaire‐based survey

## Abstract

**Objective:**

The diagnosis of childhood‐onset cerebellar ataxia (CA) is often challenging due to variations in symptoms and etiologies. Despite the known regional differences in the prevalence of etiologies underlying CA, the frequency and characteristics of CA in Japan remain unclear. We conducted a questionnaire‐based survey to identify the clinical characteristics of childhood‐onset CA in the Japanese population.

**Materials and Methods:**

Questionnaires were sent to 1,103 board‐certified pediatric neurologists in Japan from 2016 to 2017. The primary survey requested the number of patients with CA under care, and the follow‐up secondary questionnaire requested additional clinical characteristics of the patients.

**Results:**

The primary survey obtained 578 responses (response rate, 52.4%) on 385 patients with CA, including 171 diagnosed and 214 undiagnosed cases (diagnostic rate, 44.4%). The most frequent etiology was dentatorubropallidoluysian atrophy (DRPLA), followed by mitochondrial disorders and encephalitis. The secondary survey obtained the clinical characteristics of 252 cases (119 diagnosed and 133 undiagnosed cases). Multiple logistic regression analysis revealed that a younger age at onset, hearing issues, and short stature were associated with a higher risk of remaining undiagnosed with CA in Japan.

**Conclusions:**

The diagnostic rate of childhood‐onset CA in the current study was comparable to those reported in other countries. The high prevalence of autosomal dominant ataxia, especially DRPLA, was a signature of CA in Japan. These data offer insights into the characteristics of childhood‐onset CA in the Japanese population.

## Graphical abstract

We conducted a questionnaire‐based survey to identify the clinical characteristics of childhood‐onset cerebellar ataxia (CA) in Japan Questionnaires were sent to 1,103 board‐certified pediatric neurologists. The most frequent etiology of childhood‐onset CA was dentatorubropallidoluysian atrophy (DRPLA), followed by mitochondrial disorders and encephalitis. Fifty‐six percent of the collected cases remained undiagnosed. Multiple logistic regression analysis revealed that a younger age at onset, hearing issues, and short stature were associated with a higher risk of remaining undiagnosed with CA in Japan.

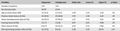

## 1 INTRODUCTION

The prevalence rates and etiologies of childhood‐onset cerebellar ataxia (CA), which defines a heterogeneous group of disorders, vary across regions (Musselman et al., 2014). For example, studies have shown that dentatorubropallidoluysian atrophy (DRPLA) is more frequent in Asia and Portugal compared to the rest of Europe and the Mediterranean (Coutinho et al., 2013; van de Warrenburg et al., 2014).

The information of childhood‐onset CA in Japan is limited. A study including both adult‐ and childhood‐onset SCAs in Japan reported that sporadic cases accounted for approximately double the number of hereditary cases and that, among hereditary SCAs, the autosomal dominant (AD) type was more common than the autosomal recessive (AR) type; this finding is contrast to that observed in the Caucasian population, in which Machado–Joseph disease/SCA3, SCA6, and DRPLA are the most common types (Tsuji, Onodera, Goto, & Nishizawa, 2008). Furthermore, Friedreich ataxia with GAA repeat expansion, the most common form of hereditary SCAs among Caucasians, has never been reported in the Japanese population (Maruyama et al., 2002). However, no studies thus far addressed childhood‐onset CA in Japan.

Due to the diverse symptoms and pathogenesis along with variations in etiologies across regions, the diagnosis of childhood‐onset CA is often challenging and might be facilitated by a better understanding of the prevalence of underlying etiologies. Thus, surveillance is necessary to update the epidemiology of childhood‐onset CA.

In the current study, we conducted a survey to collect information on childhood‐onset CA in Japan by sending questionnaires to pediatric neurologists. We also compared the clinical features between patients with and without CA diagnosis to further delineate the clinical presentation of childhood‐onset CA.

## 2 MATERIALS AND METHODS

This cross‐sectional questionnaire‐based survey was approved by the ethics committee of the National Center of Neurology and Psychiatry in Tokyo, Japan (approval number A2016‐070). The primary objective was to determine the number of patients with childhood‐onset CA in Japan and the prevalence rates of underlying etiologies. The secondary objective was to elucidate the clinical characteristics of childhood‐onset CA.

Questionnaires were sent via postal mail to 1,103 pediatric neurologists who were certified by The Japanese Society of Child Neurology as the majority of the patients with childhood‐onset CA in Japan were presumed to be under the care of these specialists. Furthermore, assessment of the neurological findings including ataxia by the pediatric neurologists was considered to be reliable.

The questionnaires were sent in two phases. The primary survey requested the number and diagnosis of patients with CA as a symptom who were under the care of the pediatric neurologist. Information on patients with CA who did not receive a definitive diagnosis was also requested. The secondary survey requested further clinical information of the patients, including disease onset, sex, family history, and clinical course (progressive or nonprogressive), but did not include personal information that could specify the individual.

The primary survey took place from November 2016 to February 2017. The secondary survey was sent to those physicians who agreed to participate in the secondary survey among those who initially responded to have at least one patient with CA under care. All data were anonymized by the responding physicians, and the answers to the questionnaires were sent back by postal mail.

The inclusion criteria were as follows: disease onset between birth and 18 years of age, presence of cerebellar atrophy or hypoplasia confirmed by magnetic resonance imaging, and presence of ataxia.

The data extracted from the questionnaires were transcribed into an excel chart and categorized into two groups: one group comprising patients without a definitive diagnosis (undiagnosed group) and one group comprising patients with a diagnosis of a specific disorder (diagnosed group). The number of patients with specific diagnoses was determined in the diagnosed group.

In addition, multiple logistic regression analysis was performed to assess the ability of independent variables among symptoms, signs, and disease course to predict the definitive diagnosis of CA in the standard medical setting of Japan. A stepwise approach was adopted for variable selection with inclusion and exclusion of criteria with a *p* value of .15. The C‐statistic and the Hosmer–Lemeshow test were used to measure the goodness of fit for the logistic regression model. A *p* value of <.05 was considered as statistically significant in all analyses. Statistical analysis was performed with SAS ver. 9.4 (SAS Institute).

## 3 RESULTS

**Figure 1:**
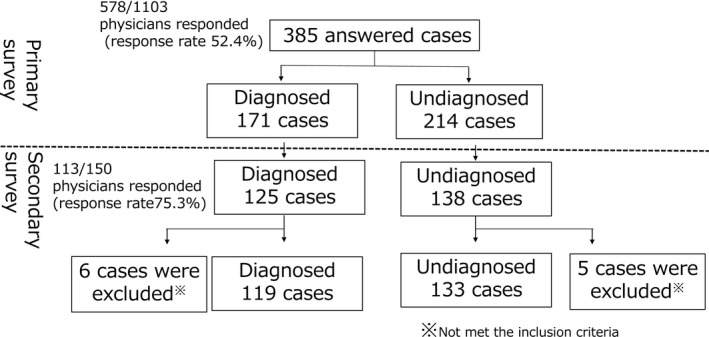
The flowchart of data collected via the primary and secondary survey. Questionnaires were sent to 1,103 board‐certified pediatric neurologists in Japan. The response rates were 52.3% in the primary survey and 75.3% in the secondary survey. A total of 385 cases, including 171 diagnosed and 214 undiagnosed cases, with cerebellar ataxia were reported, with a diagnostic rate of 44.4%

Figure 1 describes the flowchart for data collection via the primary and secondary surveys. A total of 578 replies were obtained from 1,103 requests sent during the primary survey (response rate 52.4%). Of the 578 physicians who replied, 150 stated that they had patients with childhood‐onset CA under care, which yielded information on a total of 385 cases. Therefore, it was speculated that between 150/1,103 (13.6%) and 150/578 (26.0%) of the pediatric neurologists in Japan were engaged in the treatment of childhood‐onset CA. The cases collected via the primary survey included 171 and 214 patients with and without diagnosis, respectively, indicating a diagnostic rate of 44.4% for childhood‐onset CA.

**Figure 2:**
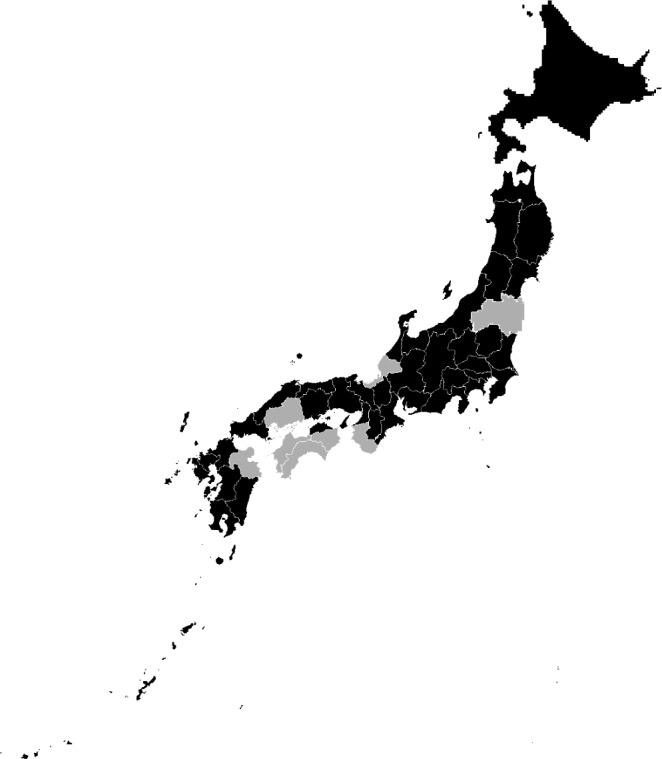
The prefectures of Japan which the responses were obtained. Black indicates at least one patient was present in the prefecture, and gray indicates responses were obtained, but 0 patient present. Gray prefectures are Fukushima, Fukui, Hiroshima, Tokushima, Wakayama, Kochi, Ehime, and Oita, from north to south

**Figure 3:**
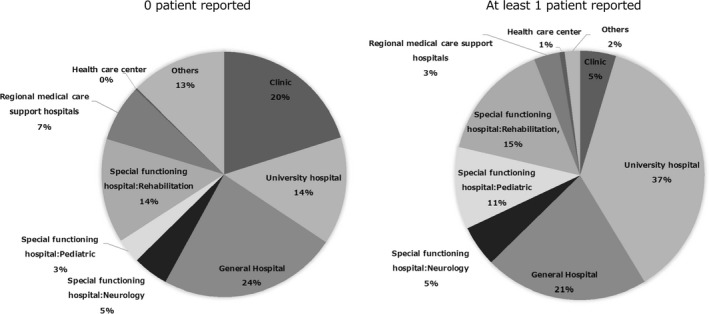
The charts showing the types of medical facilities which the responses were obtained. The facilities with no patient included more clinics, and lower number of university hospitals compared to the facilities with at least one patient. The majority of the facility with at least one patient consisted of university hospitals, general hospitals, and special functioning hospitals

The collected responses covered all of the 47 prefectures in Japan, with at least one patient reported in 39 prefectures and zero patients reported in eight prefectures (Fukushima, Fukui, Hiroshima, Tokushima, Wakayama, Kochi, Ehime, and Oita; Figure 2). The responses were obtained from university hospitals, general hospitals, special functioning hospitals (neurology, pediatrics, or rehabilitation), regional medical care support hospitals, clinics, healthcare centers, and others (included research institutes and unanswered). The majority of the reported patients were under care of one of the following: university hospital, general hospital, or special functioning hospital (Figure 3).

In the secondary survey sent to the 150 pediatric neurologists, detailed clinical information on 263 patients, including 125 diagnosed and 138 undiagnosed patients, was obtained from 113 physicians responding to the survey (response rate, 75.3%). After examination by the authors, 11 patients, including six diagnosed and five undiagnosed patients, were excluded because they did not fulfill the inclusion criteria of the current study. Therefore, information on 252 patients, including 119 diagnosed and 133 undiagnosed patients, was included in the final analysis. The patients' current age ranged from 1 to 55, and there were 201 individuals who were under age 20.

Table 1 summarizes the diagnoses of the patients with childhood‐onset CA identified via the primary survey. Of the 171 diagnosed cases obtained by the primary survey, the most frequent diagnosis was DRPLA (*n* = 30), followed by mitochondrial disorders (*n* = 20), encephalitis (*n* = 16), Joubert syndrome and related disorders (*n* = 14), and ataxia telangiectasia (AT) and related disorders (*n* = 14).

**Table 1:** Summary of the 171 diagnosed cases collected via primary survey

Diagnosis	Number of patients	Disease category
Dentatorubropallidoluysian atrophy	30	SCAs
Mitochondrial disorder	20	Inborn error of metabolism
Encephalitis	16	Encephalitis
Joubert syndrome and related disorder	14	Joubert syndrome and related disorder
Ataxia telangiectasia and related disorders	14	SCAs
Dandy–Walker variants	5	Brain malformation
Ataxia with oculomotor apraxia type 1	5	SCAs
SCA29	4	SCAs
*CASK* mutation	4	Other genetic disorders
*KIF1A* mutation	4	Other genetic disorders
Opsoclonus myoclonus syndrome	4	Paraneoplastic cerebellar degeneration
Spinocerebellar degeneration	3	SCAs
Congenital disorder of glycosylation	3	Inborn error of metabolism
*CACNA1A* mutation	3	Other genetic disorders
H‐ABC	3	Degenerative disorders
Cerebellar hypoplasia	2	Brain malformation
SCA5	2	SCAs
Peroxisomal biogenesis disorders	2	Inborn error of metabolism
Glut1 deficiency	2	Inborn error of metabolism
*SEPSECS* mutation	2	Pontocerebellar hypoplasia
Phenytoin side effect	2	Others
Cerebellar vermis deficiency	1	Brain malformation
Chiari malformation	1	Brain malformation
Holoprosencephaly	1	Brain malformation
18 trisomy	1	Chromosomal and congenital abnormality
21 trisomy	1	Chromosomal and congenital abnormality
5p syndrome	1	Chromosomal and congenital abnormality
Chromosomal abnormality	1	Chromosomal and congenital abnormality
Partial monosomy 13	1	Chromosomal and congenital abnormality
Phelan–McDermid syndrome	1	Chromosomal and congenital abnormality
Cockayne syndrome	1	Chromosomal and congenital abnormality
Pontocerebellar hypoplasia type 7	1	Pontocerebellar hypoplasia
Marinesco–Sjögren syndrome	1	SCAs
SCA2	1	SCAs
Infantile‐onset spinocerebellar degeneration	1	SCAs
L‐2‐hydroxyglutaric aciduria	1	Inborn error of metabolism
Congenital GPI deficiency	1	Inborn error of metabolism
*KMT2B* mutation	1	Other genetic disorders
*STXBP1* mutation	1	Other genetic disorders
*PLA2G6*‐associated neurodegeneration	1	Other genetic disorders
*MECP2* duplication	1	Other genetic disorders
Ceroid lipofuscinosis neuronal 6	1	Other genetic disorders
MTHFR deficiency	1	Other genetic disorders
4H syndrome	1	Degenerative disorders
Infantile neuroaxonal dystrophy	1	Degenerative disorders
Chédiak‐Higashi syndrome	1	Abnormal immune system
Post‐head trauma	1	Others
Cerebral palsy	1	Others

Abbreviations: 4H syndrome, hypomyelination with hypogonadotropic, hypogonadism and hypodontia syndrome; GPI, glucose phosphate isomerase; H‐ABC, hypomyelination with atrophy of the basal ganglia and cerebellum; MTHFR, methylenetetrahydrofolate reductase; SCA, spinocerebellar ataxia.

There were 61 patients with spinocerebellar ataxias (SCAs). Among these, the AD SCAs (*n* = 37) were more predominant than the AR SCAs (*n* = 21). The most frequent AD SCA was DRPLA (*n* = 30), followed by SCA29 (*n* = 4). Among the AR SCAs, AT and related disorder (*n* = 14) were the most common, followed by ataxia with oculomotor apraxia type 1 (AOA1; *n* = 5). Other AR SCAs such as Marinesco–Sjögren syndrome and infantile‐onset spinocerebellar degeneration, albeit observed, were less common.

Among the mitochondrial diseases, there were Leigh encephalopathy (*n* = 5); myoclonus epilepsy associated with ragged‐red fibers (*n* = 3); pyruvate dehydrogenase complex deficiency (*n* = 3); mitochondrial myopathy, encephalopathy, lactic acidosis, and stroke‐like episodes (*n* = 2); chronic progressive external ophthalmoplegia (*n* = 1); neuropathy, ataxia, and retinitis pigmentosa (*n* = 1); and other nonspecified mitochondrial disorders (*n* = 5); respectively.

The secondary survey provided more detailed information, and 119 diagnosed patients (Table A1 in Appendix 1) identified via the secondary survey were analyzed to elucidate age of onset for childhood‐onset CA in Japan (Table 2). Briefly, among the 119 patients, the ages of disease onset were <1 year (infantile‐onset), 1–5 years (early childhood‐onset), and >6 years (middle childhood to adolescence onset) in 56 (48.3%), 34 (29.3%), and 26 (22.4%) patients, respectively, whereas information on age at onset was missing in three patients. In the infantile‐onset group, the most common underlying disease was Joubert syndrome and related disorders (*n* = 11), followed by SCA29 (*n* = 4), mitochondrial diseases (*n* = 4), and *CASK*‐related disorders (*n* = 4). In the early childhood‐onset group, the most common etiology was DRPLA (*n* = 8), followed by AT (*n* = 7), and mitochondrial diseases (*n* = 7). In the middle childhood to adolescent‐onset group, the most common diagnosis was DRPLA as well (*n* = 8), followed by encephalitis (*n* = 5). Of note, among the patients with the frequently observed hereditary diseases, the mean ages at onset were 6.4 (±5.0), 0.2 (±0.20), 2.5 (±1.6), and 3.9 (±4.0) years for DRPLA, SCA29, AT and related disorder, and AOA1, respectively.

**Table 2:** Summary of the clinical features with 119 diagnosed cases collected via secondary survey

Diagnosis	*N*	Onset						Family history				Clinical course		
		<1 year		1−5 year		≥6 year		Yes		No		Progressive		Nonprogressive
Dentatorubropallidoluysian atrophy	20	3	8	8	15	4	18	2
Mitochondrial disorder	14	4	7	2	3	11	7	7
Joubert syndrome and related disorder	12	11	1		5	7		12
Ataxia telangiectasia and related disorders	11	3	7	1	2	9	9	2
Encephalitis	6		1	5		6	1	5
Ataxia with oculomotor apraxia type 1	5	2	1	2		5	5	
SCA29	4	4			1	3		4
*CASK* mutation	4	4				4		4
*CACNA1A* mutation	4	2	1	1		4	1	3
Glut1 deficiency	3	3			3		1	2
*KIF1A* mutation	3	2	1			3		3
Dandy–Walker variants	2	2				2		2
*SEPSECS* mutation	2		1	1		2	1	1
Congenital disorder of glycosylation	2	2			1	1		2
Marinesco–Sjögren syndrome	2	1		1		2	1	1
Peroxisomal biogenesis disorders	2		2		2		1	1
*PLA2G6*‐associated neurodegeneration	2		2		2		2	
*TUBA1A* mutation	1	1				1		1
Cerebellar hypoplasia	1	1				1		1
Holoprosencephaly	1	1				1		
Pontocerebellar hypoplasia type 7	1	1						1
Chromosome 22 partial duplication	1	1				1		1
Cockayne syndrome	1	1				1		1
Phelan–McDermid syndrome	1	1				1		1
Phenytoin side effect	1			1		1		1
Opsoclonus myoclonus syndrome	1		1			1		1
SCA5	1	1				1		1
SCA13	1		1			1		1
SCA2	1				1		1	
Infantile‐onset spinocerebellar degeneration	1	1				1		1
Friedreich ataxia^a^	1			1		1	1	
Spinocerebellar degeneration	1	1				1	1	
Multiple system atrophy	1			1		1	1	
*KCNA2* mutation	1	1				1	1	
*LONP1* mutation	1			1		1	1	
*MECP2* duplication	1	1					1	
*STXBP1* mutation	1	1				1		1
Chédiak‐Higashi syndrome	1					1		1

Abbreviation: SCA, spinocerebellar ataxia.

a Not confirmed by gene analysis.

Assessment of the 119 cases with a focus on family history revealed that 35 patients (30.2%) had a positive family history, whereas the remaining 81 patients (69.8%) had a negative family history in the diagnosed group. No information on family history was available for three patients. Among those with a positive family history, DRPLA (*n* = 15) was the most common, whereas mitochondrial diseases (*n* = 11), AT (*n* = 9), and Joubert syndrome (*n* = 7) were the most common among the patients with a negative family history (Table 2).

Among the diagnosed patients in the secondary survey (*n* = 119), 54 patients (45.4%) exhibited a progressive clinical course, 64 patients (53.8%) exhibited a nonprogressive course; there was one patient with missing information on their clinical course. Of the cases with the progressive course, the most frequent etiologies were DRPLA (*n* = 18), AT and related disorder (*n* = 9), and mitochondrial diseases (*n* = 7). In contrast, among the patients with a nonprogressive course, the most common cause was Joubert syndrome and related disorders (*n* = 12), followed by mitochondrial diseases (*n* = 7) and encephalitis (*n* = 5; Table 2).

The common symptoms and signs associated with CA in 252 patients for whom data were obtained via the secondary survey were intellectual disability (76.2%), followed by motor developmental delay (52.8%), seizures (31.3%), hypotonia (28.6%), increased deep tendon reflexes (26.2%), ophthalmologic problems (19.8%), muscle weakness (19.0%), and involuntary movements (18.7%).

Multiple logistic regression analysis of the 252 patients in the secondary survey, performed to identify predictive variables for definitive diagnosis, demonstrated that age onset (odds ratio [OR] 1.09, 95% confidence interval [CI] 1.01–1.18, *p* = .03), hearing issues (OR 0.07, 95%CI 0.01–0.88, *p* = .04), short stature (OR 0.32, 95%CI 0.12–0.82, *p* = .02), and decreased deep tendon reflexes (OR 6.38, 95%CI 2.03–20.1, *p* < .01) were significantly associated with definitive diagnosis for childhood‐onset CA in Japan (Table 3). Albeit not statistically significant, negative family history and lack of motor developmental delays tended to be associated with failure of a definitive diagnosis (Table 3). The C‐statistics was 0.70, and the *p* value of Hosmer‐Lemeshow test was .76, indicating a good model fitting.

**Table 3:** Multiple logistic regression analysis on clinical features of 252 diagnosed and undiagnosed cases collected via secondary survey

Variables	Diagnosed	Undiagnosed	Odds ratio	Lower CL	Upper CL	*p* Value
Number of patients	119	133				
Sex [female:male]	60:59	67:66				
Age at onset [mean (*SD*)]	3.4 (4.2)	2.3 (3.5)	1.09	1.01	1.18	.03
Positive family history [*N* (%)]	35 (29.4)	25 (18.8)	1.92	1.00	3.69	.05
Motor developmental delay [*N* (%)]	66 (55.5)	67 (50.4)	1.67	0.92	3.01	.09
Hearing issues [*N* (%)]	2 (1.7)	5 (3.8)	0.07	0.01	0.88	.04
Short stature [*N* (%)]	8 (6.7)	22 (16.5)	0.32	0.12	0.82	.02
Decreased deep tendon reflex [*N* (%)]	21 (17.6)	8 (6.0)	6.38	2.03	20.10	<.01

Note

Odds ratios for other than “age at onset” are described as “yes/no”.

Sex was not selected with the stepwise procedure in the logistic model.

Abbreviations: CL, confidence limit; *SD*, standard deviation.

We further examined diagnostic tests that contributed to definitive diagnosis and found that genetic analysis, brain imaging, and testing of body fluids (blood and cerebrospinal fluid) were required in 73.1%, 49.6%, and 17.6% of the patients with a definitive diagnosis of childhood‐onset CA, respectively. Of note, multiple test modalities were necessary to confirm the diagnosis in some patients.

Among the undiagnosed cases, 45 patients underwent genetic analyses including whole‐exome sequencing (WES; *n* = 10) and disease‐specific genetic analysis (*n* = 14). No precise information regarding genetic analyses was available for the remaining 21 patients.

The most common brain imaging finding that contributed to the diagnosis was molar tooth sign (vermian hypoplasia) in Joubert syndrome and related disorders. Other brain imaging findings which contributed to the diagnosis in the diagnosed group were diffuse brain atrophy in DRPLA, basal ganglia abnormalities in mitochondrial diseases, calcification of the basal ganglia in Cockayne syndrome, and vermian atrophy with enlarged posterior fossa in Dandy–Walker variant. Among the tests for body fluids, elevated lactate and pyruvic acid in mitochondrial diseases, elevated alpha‐fetoprotein, and decreased immunoglobulin A in AT were described as contributory to the diagnosis. Other findings such as giant granules in white blood cells in Chédiak‐Higashi syndrome and low blood glucose level in cerebrospinal fluid in GLUT1 deficiency were also reported as contributors to the diagnosis. Abnormal findings in transferrin electrophoresis were contributory to the diagnosis of carbohydrate‐deficient glycoprotein syndrome type 1a.

## 4 DISCUSSION

The current study demonstrated that definitive diagnosis was achieved in 44.4% of the patients with childhood‐onset CA in Japan, with the 55.6% of the patients remaining undiagnosed; this finding is in agreement with previously published diagnostic rate of 47% in 2012 from Canada (Al‐Maawali, Blaser, & Yoon, 2012). Among the patients with a definitive diagnosis, DRPLA, mitochondrial diseases, encephalitis, AT and related disorders, and Joubert syndrome and related disorders were the most common etiologies of childhood‐onset CA in Japan.

Given that the population of Japanese children younger than 20 years is 21,580,000 (e‐Stat, 2019, January 20) and that 201 patients younger than 20 years were included in the current study, the estimated overall prevalence of children with CA in Japan is at least 0.93 per 100,000. Systematic reviews and meta‐analyses to assess the frequency of childhood‐onset CA reported the overall prevalence of ataxia in Europe was no more than 26 per 100,000 children (Musselman et al., 2014), which is higher than our findings, which might be attributable to differences in prevalence rates across countries or differences in methods used across studies.

Studies suggest the significance of age at onset, clinical progression (progressive or nonprogressive), and family history for the definitive diagnosis of childhood‐onset CA (Fogel & Perlman, 2007; Singer, Mink, Gilbert, & Jankovic, 2015). The average age at onset for AT in the current study (2.5 ± 1.6 years) is lower than the previously reported onset age of above 5 years (Singer et al., 2015). Furthermore, the onset age for AOA1, which follows a similar clinical course as AT, was reported as approximately 7 years (Fogel & Perlman, 2007), which was 3.9 ± 4.0 years in the current study, indicating that the onset of AT and AOA1 might be earlier in the Japanese population. DRPLA is a well‐known AD SCA with an early childhood‐onset (Fogel, 2012; Singer et al., 2015). Studies assessing adult‐onset as well as childhood‐onset DRPLA reported the age of onset for DRPLA as 36.9 ± 18.0 years in Japan (Al‐Maawali et al., 2012; Maruyama et al., 2002); however, the current study indicated that the onset age for childhood‐onset DRPLA was 6.4 ± 5.0 years.

Assessment of the clinical progression in the study cohort revealed that SCAs were the most common etiology among patients with a progressive course, whereas Joubert syndrome and related disorders was the most common cause among the nonprogressive cases. Previous studies implicated that ataxias with congenital or nonspecific causes, including cerebral palsy, tended to exhibit a static course; an acute progressive course might result from intoxications, infections, or inflammatory processes; and chronic progressive ataxia might result from genetic disorders (Fogel, 2012; Singer et al., 2015). These findings are in agreement with the current study findings, suggesting that genetic disorders should be considered in chronic progressive cases and that congenital malformations should be included in the differential diagnosis for nonprogressive cases in patients with childhood‐onset CA in Japan. However, it should be noted that certain genetic disorders with extremely slow progression might be misdiagnosed as nonprogressive CA.

Among the SCAs with a positive family history, DRPLA was the most common cause; however, there were four patients with DRPLA who had no family history even though a positive family history is a common feature of AD SCAs. In the current study, SCA29 (*n* = 4) was the second most common cause of AD SCAs, with three patients exhibiting no family history.

In the current study, the mean onset age of 2.3 ± 3.5 years and positive family history rate of 18.8% within the undiagnosed group were similar to those reported in cohorts from Canada, demonstrating that the majority (82%) of undiagnosed cases presented at ages younger than 2 years and comprised a smaller population (19%) with a positive family history (Al‐Maawali et al., 2012).

Multiple regression analysis using clinical features as variables indicated that younger age of onset, hearing issues, and short stature were risk factors for not receiving a definitive diagnosis for childhood‐onset CA in Japan. Patients with *C10ORF2* mutation, pontocerebellar hypoplasia type 7, Marinesco–Sjögren syndrome, DRPLA, *MECP2* mutation, or mitochondrial disorders were included in the diagnosed individuals who had young age onset, with either hearing issue or short stature. Therefore, these disorders may be first considered as differential diagnosis if not have been assessed. Further, AR SCAs are generally associated with clinical findings outside the nervous system (Fogel & Perlman, 2007; Singer et al., 2015). Hearing issues and a short stature might be unrelated to the nervous system, and the undiagnosed cohort of the current study might have included patients with AR SCAs. Another speculation might be that individuals with childhood‐onset CA harboring hearing issues or a short stature might be representing a new disease entity with these signs that is yet to be identified.

In AD SCAs, genetic panel testing screening is a mainstream diagnostic approach for SCA types 1, 2, 3, 6, 7, and 17, and DRPLA (van de Warrenburg et al., 2014). Several recent studies demonstrated the utility of WES for the diagnosis of childhood‐onset CA, especially AR SCAs, a rapidly expanding group following the implementation of this technology (Fogel, 2012; Ohba et al., 2013; Sasaki et al., 2015; Sawyer et al., 2014; van de Warrenburg et al., 2014). Given the possibility that there might be patients with AR ataxias in the undiagnosed group, the diagnostic rate might improve with the implementation of WES for all patients with no definitive diagnosis.

One notable limitation of the current study was the administration of the survey to a limited number of physicians and facilities. Nonetheless, to best of our knowledge, this is the first study to investigate patients with childhood‐onset CA in Japan, providing new insights on the prevalence and etiologies and the feasibility of potential predictors of diagnosis in these patients.

## CONFLICT OF INTEREST

The authors have no conflict of interests to declare.

## Data Availability

The data that support the findings of this study are available from the corresponding author upon reasonable request.

## Appendix 1

**Table A1:** Summary of the 119 diagnosed cases collected via secondary survey

Diagnosis	Number of patients	Disease category
Dentatorubropallidoluysian atrophy	20	SCAs
Mitochondrial disorder	14	Inborn error of metabolism
Joubert syndrome and related disorder	12	Joubert syndrome and related disorder
Ataxia telangiectasia and related disorders	11	SCAs
Encephalitis	6	Encephalitis
Ataxia with oculomotor apraxia type 1	5	SCAs
SCA29	4	SCAs
*CASK* mutation	4	Other genetic disorders
*CACNA1A* mutation	4	Other genetic disorders
Glut1 deficiency	3	Inborn error of metabolism
*KIF1A* mutation	3	Other genetic disorders
Dandy–Walker variants	2	Brain malformation
*SEPSECS* mutation	2	Pontocerebellar hypoplasia
Congenital disorder of glycosylation	2	Inborn error of metabolism
Marinesco–Sjögren syndrome	2	SCAs
Peroxisomal biogenesis disorders	2	Inborn error of metabolism
*PLA2G6*‐associated neurodegeneration	2	Other genetic disorders
*TUBA1A* mutation	1	Brain malformation
Cerebellar hypoplasia	1	Brain malformation
Holoprosencephaly	1	Brain malformation
Pontocerebellar hypoplasia type 7	1	Pontocerebellar hypoplasia
Chromosome 22 partial duplication	1	Chromosomal and congenital abnormality
Cockayne syndrome	1	Chromosomal and congenital abnormality
Phelan–McDermid syndrome	1	Chromosomal and congenital abnormality
Phenytoin side effect	1	Others
Opsoclonus myoclonus syndrome	1	Paraneoplastic cerebellar degeneration
SCA5	1	SCAs
SCA13	1	SCAs
SCA2	1	SCAs
Infantile‐onset spinocerebellar degeneration	1	SCAs
Friedreich ataxia^a^	1	SCAs
Spinocerebellar degeneration	1	SCAs
Multiple system atrophy	1	SCAs
*KCNA2* mutation	1	Other genetic disorders
*LONP1* mutation	1	Other genetic disorders
*MECP2* duplication	1	Other genetic disorders
*STXBP1* mutation	1	Other genetic disorders
Chédiak‐Higashi syndrome	1	Abnormal immune system

Abbreviation: SCA, spinocerebellar ataxia.

a Not confirmed by gene analysis.
